# Accuracy and reproducibility of semi-automated late gadolinium enhancement quantification techniques in patients with hypertrophic cardiomyopathy

**DOI:** 10.1186/s12968-014-0085-x

**Published:** 2014-10-07

**Authors:** Yoko Mikami, Louis Kolman, Sebastien X Joncas, John Stirrat, David Scholl, Martin Rajchl, Carmen P Lydell, Sarah G Weeks, Andrew G Howarth, James A White

**Affiliations:** Stephenson Cardiac Imaging Centre at the Libin Cardiovascular Institute of Alberta, University of Calgary, Calgary, AB Canada; Imaging Research Laboratory - Robarts Research Institute, Western University, London, ON Canada; Diagnostic Imaging, University of Calgary, Calgary, AB Canada; Cardiac Sciences, University of Calgary, Calgary, AB Canada

**Keywords:** Hypertrophic cardiomyopathy, Cardiovascular magnetic resonance, Late gadolinium enhancement

## Abstract

**Background:**

The presence and extent of late gadolinium enhancement (LGE) has been associated with adverse events in patients with hypertrophic cardiomyopathy (HCM). Signal intensity (SI) threshold techniques are routinely employed for quantification; Full-Width at Half-Maximum (FWHM) techniques are suggested to provide greater reproducibility than Signal Threshold versus Reference Mean (STRM) techniques, however the accuracy of these approaches versus the manual assignment of optimal SI thresholds has not been studied. In this study, we compared all known semi-automated LGE quantification techniques for accuracy and reproducibility among patients with HCM.

**Methods:**

Seventy-six HCM patients (51 male, age 54 ± 13 years) were studied. Total LGE volume was quantified using 7 semi-automated techniques and compared to expert manual adjustment of the SI threshold to achieve optimal segmentation. Techniques tested included STRM based thresholds of >2, 3, 4, 5 and 6 SD above mean SI of reference myocardium, the FWHM technique, and the Otsu-auto-threshold (OAT) technique. The SI threshold chosen by each technique was recorded for all slices. Bland-Altman analysis and intra-class correlation coefficients (ICC) were reported for each semi-automated technique versus expert, manually adjusted LGE segmentation. Intra- and inter-observer reproducibility assessments were also performed.

**Results:**

Fifty-two of 76 (68%) patients showed LGE on a total of 202 slices. For accuracy, the STRM >3SD technique showed the greatest agreement with manual segmentation (ICC = 0.97, mean difference and 95% limits of agreement = 1.6 ± 10.7 g) while STRM >6SD, >5SD, 4SD and FWHM techniques systematically underestimated total LGE volume. Slice based analysis of selected SI thresholds similarly showed the STRM >3SD threshold to most closely approximate manually adjusted SI thresholds (ICC = 0.88). For reproducibility, the intra- and inter-observer reproducibility of the >3SD threshold demonstrated an acceptable mean difference and 95% limits of agreement of −0.5 ± 6.8 g and −0.9 ± 5.6 g, respectively.

**Conclusions:**

FWHM segmentation provides superior reproducibility, however systematically underestimates total LGE volume compared to manual segmentation in patients with HCM. The STRM >3SD technique provides the greatest accuracy while retaining acceptable reproducibility and may therefore be a preferred approach for LGE quantification in this population.

## Background

Late gadolinium enhanced (LGE) cardiovascular magnetic resonance (CMR) has the capacity to identify regional accumulation of myocardial fibrosis in patients with hypertrophic cardiomyopathy (HCM) [1-3]. This imaging biomarker is identified in approximately two-thirds of patients with HCM and has been associated with adverse outcomes [4,5]. However, given the high prevalence of this finding when reported as a binary variable, accurate and reproducible methodologies aimed at LGE burden quantification are required to more adequately estimate risk among this population.

In contrast to ischemic injury, the fibrosis associated with HCM is typically patchy and non-uniform, posing a substantial challenge for manual segmentation. The visual application of a circumferential boundary is therefore an inappropriate reference standard in this population, particularly when being used to validate voxel-based threshold techniques. Despite this, studies to date have largely used manual planimetry as a reference when testing semi-automated LGE segmentation. Such techniques described to date include; i) Signal Threshold versus Reference Mean (STRM) [1,4,6], ii) Full Width at Half Maximum (FWHM) [7], and the Otsu Auto Threshold (OAT) techniques [8]. The most notable comparative study inclusive of patients with HCM by Flett et al. included 20 patients with previously established diagnosis. In this study, they reported that the FWHM technique resulted in higher reproducibility metrics than for STRM [9]. However, the accuracy of these techniques to replicate an expert-based segmentation of LGE was not tested. While other studies have attempted to determine the accuracy of various STRM-based signal intensity (SI) thresholds against a visual standard [6,10], manual LGE planimetry is most commonly considered the reference standard.

In this study we systematically investigated the accuracy and reproducibility of all known semi-automated threshold-based LGE quantification techniques within a large cohort of patients with confirmed HCM. We employed a novel gold standard that represented signal enhancement by expert visual adjustment of a signal intensity threshold for each analyzed image. Based upon our findings we provide recommendations for the optimal selection of signal threshold techniques in reporting total LGE among patients with HCM.

## Methods

### Patients

Patients were identified from a prospectively enrolled clinical registry of HCM patients undergoing CMR between March 2008 and May 2011 at the Cardiovascular MRI Clinical Research (CMCR) Center at Western University, Canada. Inclusion criteria for this study were adult patients (age ≥18) with echocardiographically diagnosed HCM, defined according to AHA consensus guidelines, including the presence of a hypertrophied (≥15 mm or ≥ 13 mm if a 1st degree relative with HCM) and non dilated left ventricle in the absence of another cardiac or systemic disease capable of producing this magnitude of hypertrophy [11]. Eighteen patients were excluded from analysis due to prior surgical myomectomy or alcohol septal ablation. All patients provided written informed consent and the study protocol was approved by the Western University Research Ethics Board.

### CMR protocol

All CMR studies were performed on a clinical 3-T MRI system (TRIO or Verio, Siemens Healthcare, Erlangen, Germany) with a 32 channel cardiac coil using ECG gating. The imaging protocol involved short axis cine imaging and LGE imaging. LGE imaging was performed using inversion recovery gradient echo sequence 10 minutes after the administration of Gadolinium contrast (Magnevist® or Gadovist®, Bayer Inc, Toronto, Ontario, Canada) at a dose of 0.2 mmol/kg. Typical imaging parameters for LGE were: slice thickness 8 mm, gap 2 mm, TE 1.93 ms, flip angle 20 degrees, matrix 256 × 205, TI individually determined to minimize the SI of normal myocardium range 200 to 400 ms.

### Image analysis

All images were anonymized and analyzed in random order. Cine images were blindly analyzed using certified software (cvi^42^, Circle CVI, Calgary, Alberta, Canada) for determination of LV volume, mass and ejection fraction (EF). Papillary muscle was included as part of myocardium. Maximum wall thickness was measured from the short axis views.

**Figure 1:**
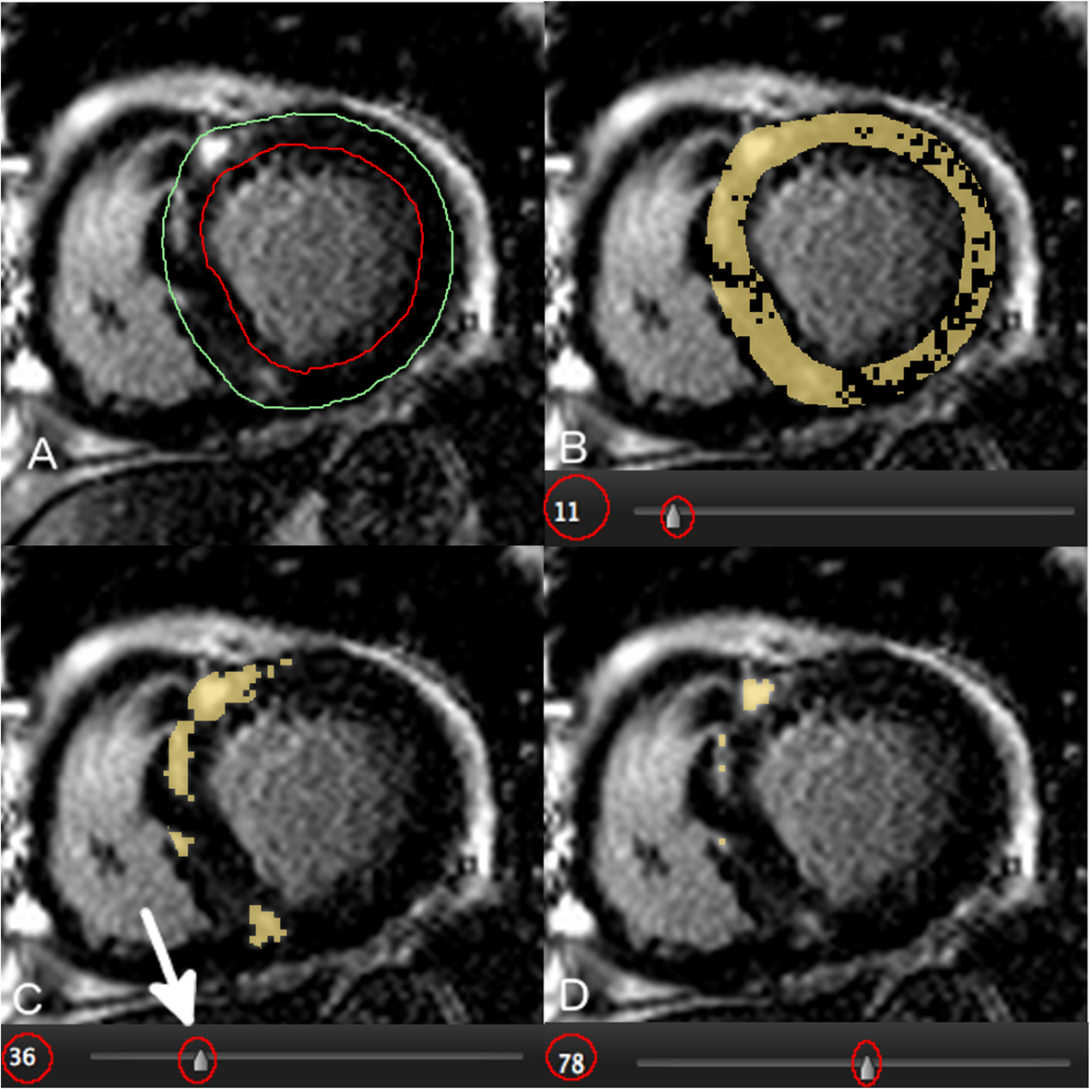
**Example of the expert Late Gadolinium Enhancement (LGE) segmentation procedure using a manual adjustment of the signal intensity (SI) threshold for each LGE image.** A manual SI threshold (circled) was adjusted using a slide bar until visually identified LGE was segmented in accordance to expert opinion. Panel **A** shows a raw LGE image prior to application of segmentation. Panel **B** shows over-representation of LGE at a low SI threshold of 11. Panel **C** shows “optimal segmentation” at a threshold of 36. Panel **D** shows under-representation of LGE at a higher threshold of 78. The signal threshold of 36 (arrow) was applied for this image and the corresponding LGE area employed as a reference standard for semi-automated technique comparison.

**Figure 2:**
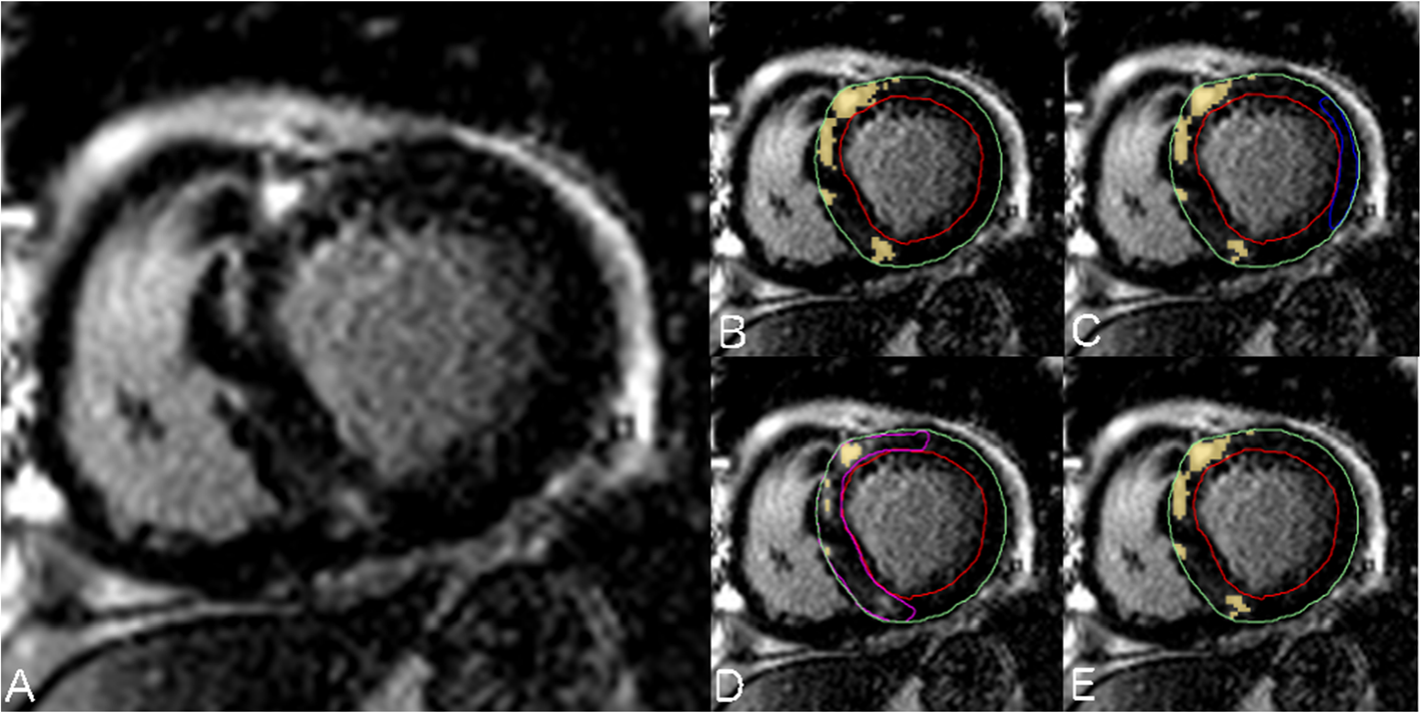
**Comparison of; A) Raw LGE image, B) Expert manual segmentation, C) Signal Threshold versus Reference Mean (STRM) threshold of ≥5SD, D), Full Width at Half Maximum (FWHM), and the E) Otsu auto-threshold (OAT) methods applied to the same imaging slice.** Reference tissue regions of interest (ROI) for remote myocardium (STRM method) and maximal signal enhancement (FWHM method) are shown in blue and pink, respectively.

LGE images were analyzed by an observer with 7 years of experience in CMR (YM) using the same software (cvi^42^, Circle CVI.). Magnitude reconstruction images were used for image analysis to maximize clinical generalizability. These were first evaluated for acceptability based upon an appropriate adjustment of the inversion time, and absence of significant motion artifact. Seven patients were excluded from the study due to unacceptable image quality. For each short axis slice, the endocardial and epidcardial boundaries were manually traced with careful attention to avoid visually apparent artifacts or blood pool. These same boundary contours were used for the testing of all LGE quantification techniques to eliminate the influence of contour tracing on threshold-based LGE quantification. Total LGE volume was then quantified using 7 semi-automated techniques and compared to an expert manual adjustment of the signal intensity threshold, as shown in Figure 1. Techniques tested included STRM-based thresholds of >2, >3, >4 > 5 and >6 SD above the mean SI of reference myocardium, the FWHM technique, and the Otsu auto-threshold (OAT) technique (Figure 2). For STRM-based assessments, the reference myocardium was defined for each slice as the largest contiguous area of myocardium with no visually apparent LGE or artifact. For FWHM-based assessments, the reference region was defined for each slice as an area inclusive of the maximum signal intensity of visually apparent LGE. The resulting SI threshold applied to define LGE for each technique was recorded. Total LGE was determined on a per-patient basis for each semi-automated technique as the sum of LGE area for each slice multiplied by the slice thickness.

The gold standard of manual threshold assignment was performed for each slice as follows; the SI threshold for definition of LGE was set to zero (100% enhancement) and then manually increased until the segmented signal visually matched the visually identified LGE on each slice (Figure 1). The manual signal threshold employed for each slice was recorded.

Inter-observer and intra-observer reproducibility testing was performed by two blinded investigators (YM and JW). This was accomplished by repeating measurements in random order for 15 randomly selected patients. For this analysis the same endocardial and epicardial contours were used, again to focus reproducibility testing on the threshold techniques themselves, inclusive of the application of reference regions.

### Statistical analysis

All values were expressed as mean ± SD. Bland Altman analysis and intraclass correlation coefficients (ICC) were reported for each semi-automated technique versus expert, manually adjusted segmentation. Intra and inter-observer reproducibility assessments were similarly performed using Bland Altman analysis and ICC. All analyses were conducted using SPSS for Macintosh, version 19.0 (SPSS, Inc., Chicago, Illinois).

## Results

A total of 83 registry patients were available for image analysis. Of these, seven were excluded due to suboptimal image quality related to motion artifact or sub-optimal adjustment of the time from inversion (TI time). This resulted in 76 patients with HCM included in the study, 51 being male and having a mean age of 54 ± 13 years. All other patient characteristics are shown in Table 1.

**Table 1:** **Patient characteristics (N = 76)**

Age (years)	54 ± 13
Male, n (%)	51 (67)
Height (m)	1.7 ± 0.1
Weight (kg)	85.5 ± 18.5
BMI (kg/m^2^)	28.6 ± 5.3
Systolic BP (mmHg)	132 ± 18
Diastolic BP (mmHg)	77 ± 11
Heart Rate	65 ± 11
NYHA heart failure class, n (%)	
Class I	48 (63)
Class II	17 (22)
Class III	9 (12)
Class IV	2 (3)
LVOT resting obstruction ≥ 30 mmHg by TTE, n (%)	18 (24)
CMR findings	
LVEDVI (ml/m^2^)	60.1 ± 12.7
LVESVI (ml/m^2^)	16.2 ± 9.1
LVSVI (ml/m^2^)	43.9 ± 10.5
LV ejection fraction (%)	74 ± 12
Indexed LV mass (g/m^2^)	89 ± 27
LV maximal wall thickness (mm)	19 ± 6
LGE positive, n (%)	52 (68)
LGE mass (g)	18 ± 20
LGE mass (% of the LV mass)	11 ± 12

Values are presented as n (%) or mean ± SD. LV mass was indexed to body surface area. NYHA class was determined by patients’ symptoms interviewed at the time of Cardiovascular MRI. LGE mass values were by manual expert segmentation using slice-based signal threshold assignment. BMI = Body Mass Index; BP = Blood Pressure; NYHA = New York Heart Association; LV = Left Ventricular; LVOT = Left Ventricular Outflow Tract; TTE = Transthoracic echocardiogram; EDVI = Endo Diastolic Volume Indexed by body surface area; ESVI = Endo Systolic Volume Indexed by body surface area; SVI = Stroke Volume Indexed by body surface area; LGE = Late Gadolinium Enhancement.

Fifty-two patients (68%) were scored as having clear visual evidence of LGE, this finding being identified on a total of 202 out of 742 (27%) available image slices. Manual expert segmentation using slice-based signal threshold assignment resulted in a mean Total LGE burden among the study population of 18.2 ± 20.4 g.

### Accuracy of semi-automated LGE quantification techniques

**Figure 3:**
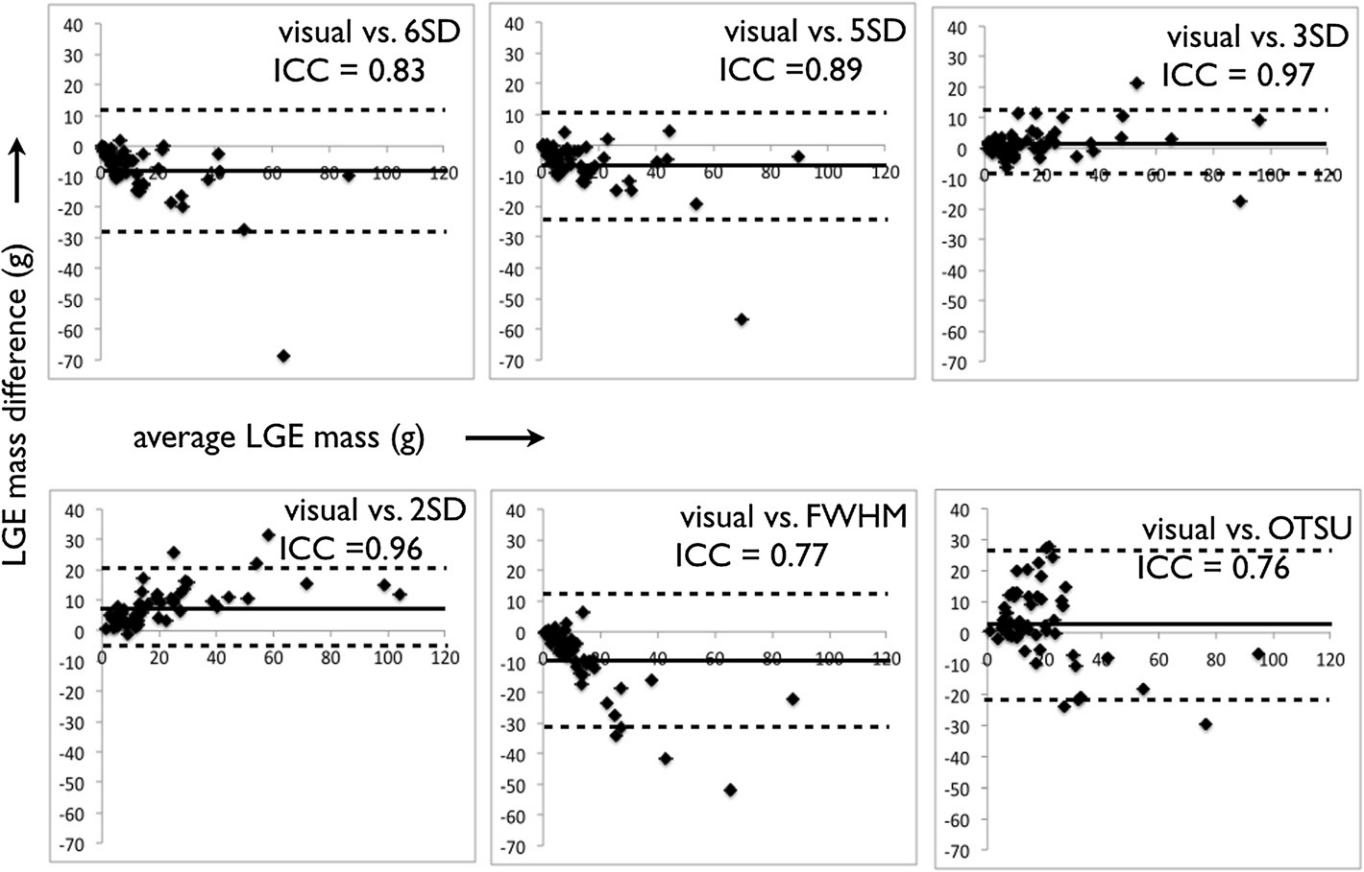
**Bland-Altman plots between each semi-automated technique and manual expert segmentation.** The Signal Threshold versus Reference Mean (STRM) >3SD technique showed greatest agreement with manual segmentation.

**Figure 4:**
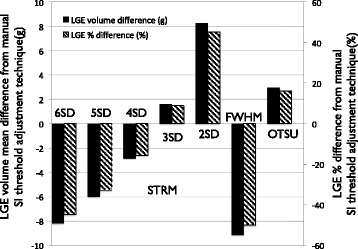
**Mean difference in Late Gadolinium Enhancement (LGE) volume (g) and % difference (%) between each semi-automated technique and expert segmentation by manual Signal Intensity (SI) threshold adjustment technique.** The Signal Threshold versus Reference Mean (STRM) >3SD technique showed the greatest agreement with manual segmentation while STRM >6SD, >5SD, >4SD and full width at half maximum (FWHM) techniques systematically underestimated total enhanced volume.

**Figure 5:**
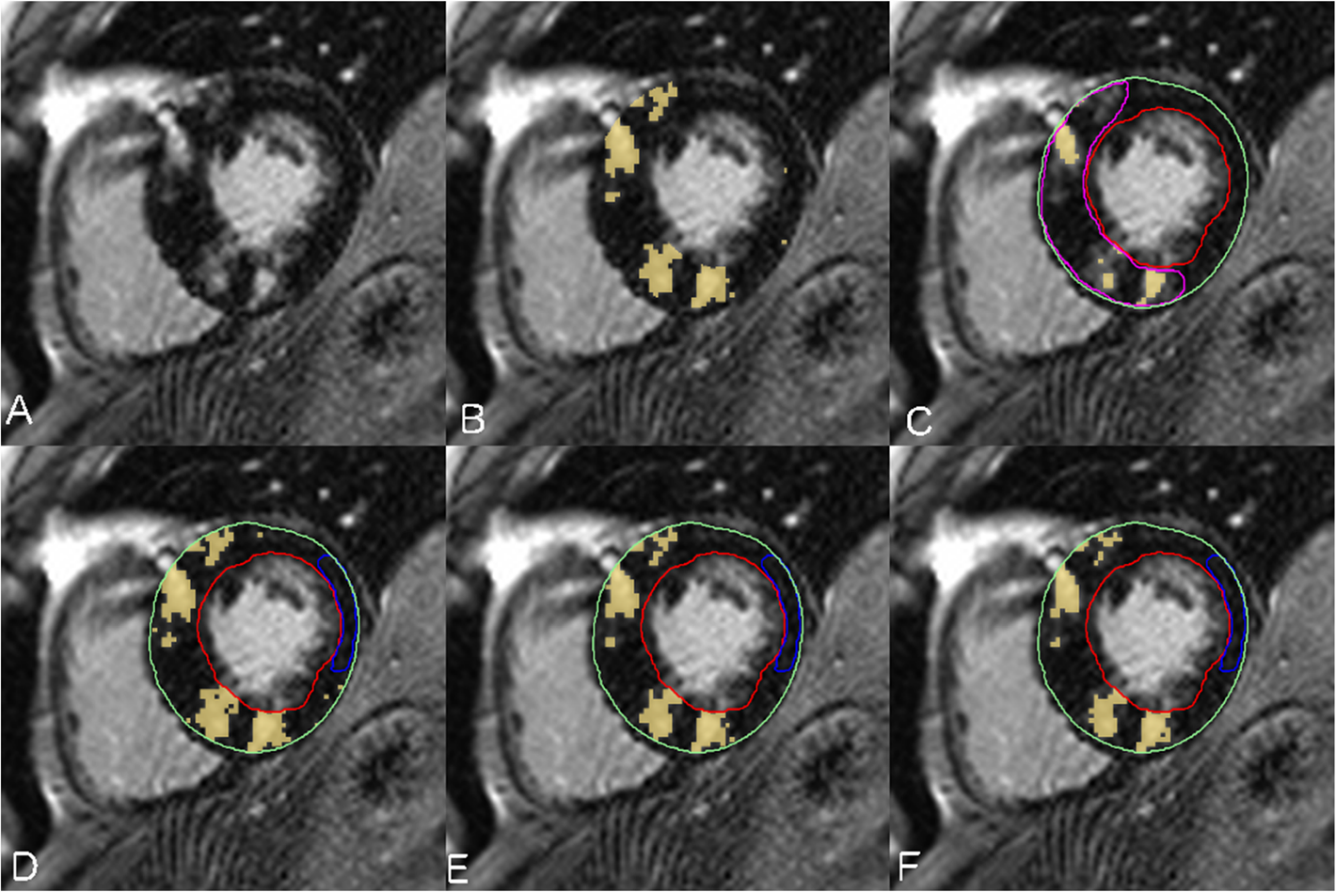
**Patient example of raw Late Gadolinium Enhanced (LGE) image (Panel A), manual expert segmentation (Panel B) versus full width at half maximum (FWHM) -based segmentation (Panel C) and the Signal Threshold versus Reference Mean (STRM) -based segmentation at >3SD (Panel D), >4SD (Panel E) and >5SD thresholds (Panel F).** A significant under-estimation of visually identifiable LGE is evident using the FWHM method.

Bland-Altman plots for the semi-automated signal threshold techniques versus expert segmentation are shown in Figure 3. Overall, the STRM >3SD technique showed the highest agreement with manual segmentation with an ICC of 0.97 (mean difference and 95% limits of agreement: 1.6 ± 10.7 g). The mean difference of total LGE burden obtained for each of the 7 semi-automated techniques versus the manually adjusted reference standard are shown in Figure 4. The STRM >6SD, >5SD, >4SD and FWHM techniques were found to systematically underestimate total LGE burden, as shown in Figures 4 and 5. Conversely, the STRM >2SD and OAT techniques were found to overestimate total LGE burden in comparison to the gold standard.

Using slice-based analysis, the SI threshold applied by each technique was compared to that obtained by manual expert threshold adjustment. This analysis similarly identified the STRM >3SD threshold to most closely approximate the manually adjusted SI threshold with an ICC of 0.88 (mean difference and 95% limits of agreement = 2.5 ± 16.8).

### Reproducibility of semi-automated LGE quantification techniques

**Figure 6:**
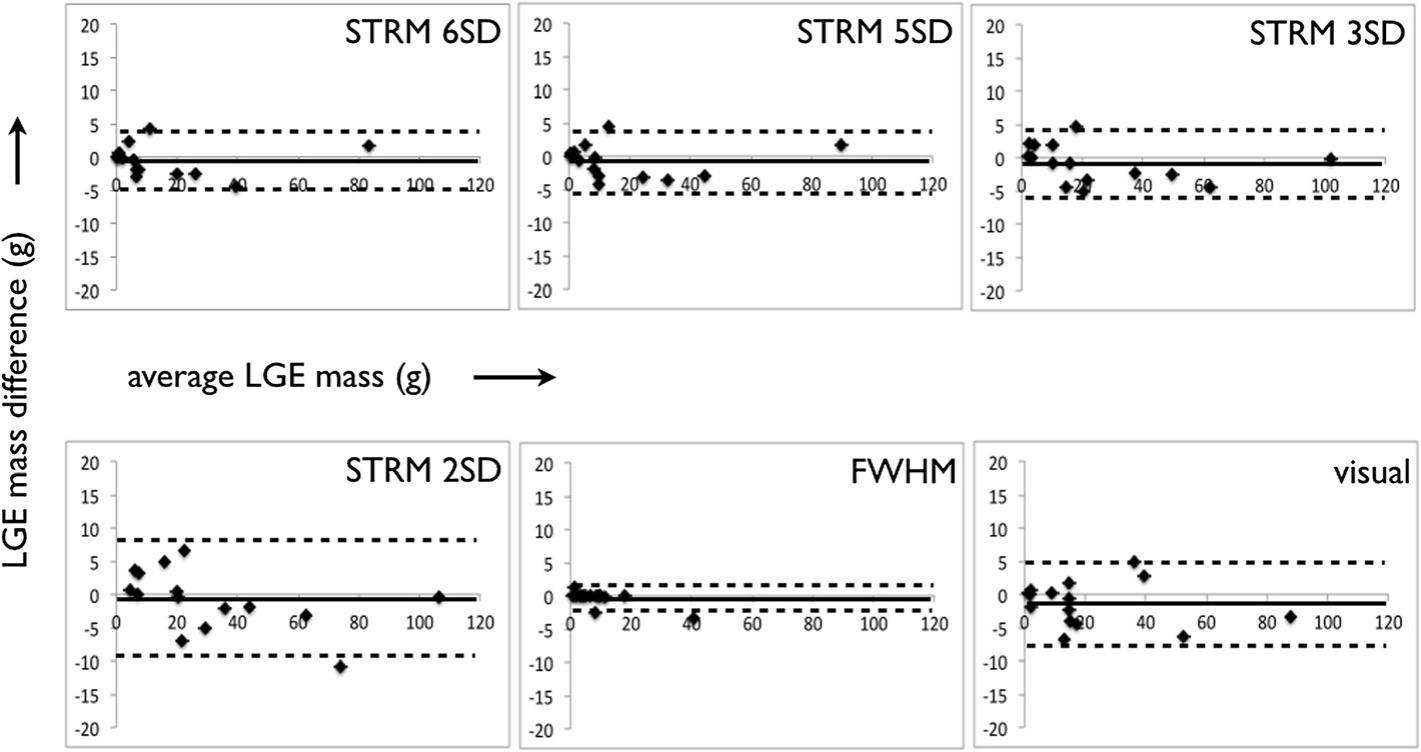
**Bland-Altman plots of** ***inter*** **-observer variability for semi-automated techniques and manual segmentation.**

**Figure 7:**
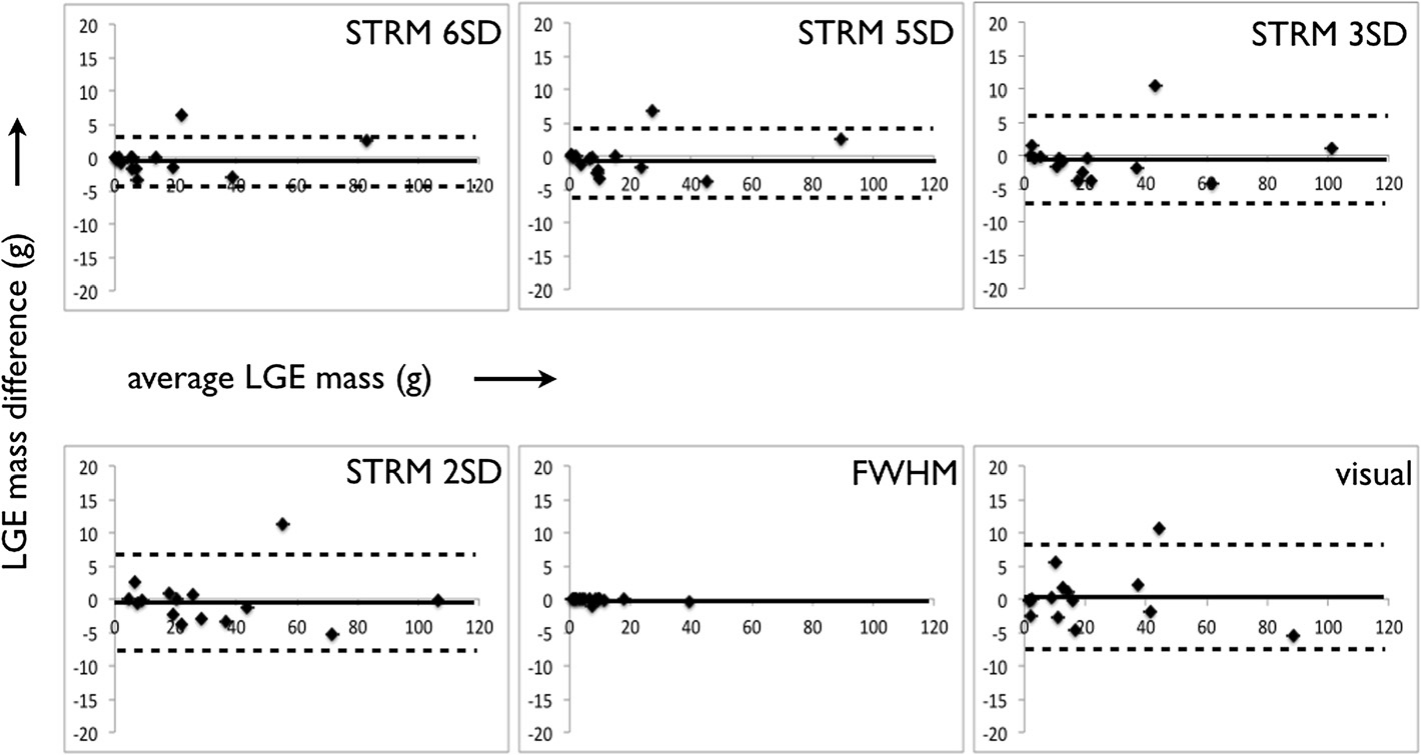
**Bland-Altman plots of** ***intra*** **-observer variability for semi-automated techniques and manual segmentation.**

Bland-Altman plots were performed for the assessment of both inter- and intra-observer variability for semi-automated segmentation techniques and for manual expert segmentation. These analyses are shown in Figures 6 and 7.

Total LGE assessment by manual signal threshold adjustment was found to be highly reproducible with a mean difference and 95% limits of agreement between observers of −1.3 ± 6.5 g, and 0.3 ± 7.8 g within the same observer. Of the semi-automated techniques, the FWHM method showed the highest intra- and inter-observer reproducibility with a mean difference and 95% limits of agreement between observers of 0.3 ± 2.2 g, and −0.1 ± 0.5 g within the same observer. Corresponding reproducibility values for STRM-based thresholds were found to improve with increasing SI thresholds (Figures 6 and 7). For example, for the intra-observer values for >2SD and >6SD thresholds were −0.3 ± 7.5 g and −0.2 ± 4.5 g while the inter-observer values were −0.7 ± 9.0 g and −0.6 ± 4.5 g, respectively.

The STRM >3SD threshold, identified among the techniques as optimal with respect to precision, demonstrated acceptable inter- and intra-observer ICC values of 0.992 and 0.995. The corresponding mean difference and 95% limits of agreement were −0.5 ± 6.8 g and −0.9 ± 5.6 g, respectively.

## Discussion

This is the largest analysis performed to date comparing both the accuracy and reproducibility of semi-automated SI threshold-based LGE quantification. In this study we focused solely on patients with HCM, a cohort recognized to pose challenge for LGE characterization due to a patchy and non-uniform distribution. Uniquely, the reference standard used in this analysis provided a more appropriate comparator of total LGE burden compared to the convention of manual planimetry.

The potential importance of LGE quantification in patients with HCM has been highlighted by a number of recent studies [4,5,12-16], each emphasizing a role for the identification of patients at elevated risk of future cardiovascular events. Despite this, only limited data is available regarding the accuracy of LGE quantification in this population [6,9]. Specifically, in contrast to ischemia-mediated injury, the distribution of collagen deposits in HCM appears highly variable in both density and distribution, ranging from diffusely dispersed patches of collagen fibers to dense and irregular islands of mature scar [1,17,18]. As such, the manual application of a linear boundary to quantify this phenomenon appears to be an inappropriate gold standard. In the current study we attempted to mitigate this limitation by employing a manually-adjusted signal threshold, a technique most congruent with the threshold-based segmentation techniques being tested. This provided a more appropriate expert-adjudicated reference standard for the assessment of accuracy.

When compared to expert opinion, our results identified the STRM > 3SD technique to provide the closest estimate of LGE burden among the population. STRM-based thresholds below this cut-off (i.e.: >2SD) systematically over-estimated LGE burden compared to the reference standard, while thresholds above this cut-off (i.e.: >4SD, >5SD and >6SD) systematically under-estimated LGE burden. The FWHM approach was shown to under-estimate LGE burden, as illustrated in Figure 4, while the OAT method systematically over-estimated LGE burden. With respect to reproducibility we identified, similar to the report by Flett et al., that the FWHM method provides superior reproducibility than STRM-based methods [9].

Based upon our findings it appears that no “ideal” segmentation technique currently exists for the quantification of LGE among patients with HCM. And, depending upon a preference towards accuracy or reproducibility, an informed decision must be made to best answer the question posed. However, when weighing the relative performance metrics of each technique, we conclude that the STRM > 3SD technique may provide the most optimal result for general use with accuracy that best represents the LGE burden identified by expert visual adjudication and provides sufficiently robust reproducibility. While the FWHM method remains attractive for reproducibility it must be used in recognition of a significant under-estimation of total LGE burden. The OAT method cannot be recommended for use in this population.

LGE has been shown to be a predictor of worse outcomes in HCM, inclusive of worse LV systolic dysfunction [12], ventricular arrhythmias [5,13,14], sudden cardiac death [13] and both all cause and cardiac mortality [4]. However, a particular challenge for its use for risk stratification in this population is a high prevalence of LGE when described as a binary finding; as high as 78% among those with genetically confirmed disease with LVH [19]. As such, attention towards quantification of LGE burden has emerged, and this is reliant upon provision of both accuracy and reproducibility. While the lack of any ideal approach has led to appropriate exploration for alternate techniques of fibrosis quantification through T1 mapping [20,21], and extra cellular volume (ECV) fraction estimation [19,22], it is anticipated that conventional LGE imaging will remain an important diagnostic and prognostic tool in clinical practice for the foreseeable future. As such, the standardization of LGE reporting in this population is required.

Several recent studies have reported on the value of volume-based quantification rather than dichotomous reporting of LGE in patients with HCM. The extent of LGE has been shown to be related to larger LV mass [23] and reduced systolic function [12,23] in this population. In a study of 217 HCM patients, the extent of LGE was associated with risk of heart failure admissions, deterioration to New York Heart Association functional class III or IV, or heart failure-related death over a mean of 3.1 years [14]. Another study assessed progression of LGE between 2 CMR examinations (mean interval of 719 days among 55 HCM patients) where a greater interval increases in LGE was associated with worsening of NYHA class [24]. Ismail et al. recently published a study of 711 HCM patients diagnosed by standard clinical criteria and followed them for a median of 3.5 years [15]. Thirty-two patients reached the primary endpoint of sudden cardiac death (SCD) or aborted SCD. The extent of LGE quantified using the FWHM method was a predictor of the primary endpoint by univariable analysis (HR per 5% LGE 1.24). However, this failed to remain predictive by multivariable analysis. The second study, published by Chan et al., included 1293 HCM patients defined by CMR findings and followed for a median of 3.3 years [16]. Thirty-seven patients experienced the primary outcomes of SCD or appropriate defibrillator therapy. The extent of LGE was quantified by a manual adjustment of gray scale threshold (similar to that used as the reference standard in the current study). Using this approach, %LGE was a significant predictor of the primary outcome in both univariate and multivariate analysis, the latter inclusive of relevant conventional risk factors (adjusted HR of 1.46/10% increase in LGE). A LGE burden ≥15% was associated with a HR of 2.14 and an estimated 5-year event rate of 6.3%. Whether differences in LGE quantification methodology are adequate to explain differences in predictive utility between the Chan et al., and Ismail et al. studies remains uncertain. However, such differences highlight a need to adopt a common, standardized approach to LGE quantification.

Appropriate debate exists, and will continue to exist, regarding optimal metrics of LGE quantification for the accuracy of cardiovascular events in specific disease cohorts. Whether a single LGE threshold is optimal to predict arrhythmic events, or whether intermediate LGE signal (ie: “border-zone”) is more discriminative among patients with ischemic cardiomyopathy remains uncertain [25-27]. This same debate could extend to the HCM population, a recent study by Appelbaum *et al.* similarly suggesting LGE signal between the range of 4SD and 6SD providing higher predictive value for non sustained ventricular arrhythmia [28]. Irrespective, we must first acknowledge fundamental strengths and weaknesses of each segmentation technique, and only then select that which is best suited to answer the question being posed.

### Study limitations

This study was designed to assess the accuracy and reproducibility of LGE quantification in patients with echocardiographically confirmed HCM at a single academic institution. Therefore, generalization of our findings beyond this referral population cannot be recommended.

We recognize that, while the employed reference standard was chosen as the best possible solution, it remains imperfect in that subjectivity is still introduced when applying manual thresholds. Histologic correlation, while desirable, was not available in this clinical cohort. However, a recently reported histologic study by Moravsky *et al.* provides complementary findings by comparing histological findings from septal myectomy samples to regionally matched LGE segmentation. While this study could not assess total LGE burden (i.e.: limited to the myomectomy sample itself), the STRM technique was similarly favoured for accurate estimates of total fibrosis burden [29]. Specifically, they found a >4SD threshold most closely approximated percent collagen deposition within the septal myomectomy sample – a threshold highly consistent with our current findings. Similar to our findings, they identified that the FWHM technique systematically under-represented histologically based measurements of fibrosis.

Finally, the process by which segmentation boundaries and reference tissues are identified is a dominant harbinger of variability for LGE quantification. The reproducibility reported in this study is based upon rigorous attention to standard operating procedures established within a core-laboratory environment and therefore represents ideal operating conditions. It is strongly advised that efforts be made to follow an established set of rules when executing LGE quantification, such as those outlined in the methods section of this study.

## Conclusions

In this large cohort study, the STRM >3SD LGE segmentation technique provided greatest accuracy and an acceptable reproducibility for total LGE burden quantification versus the reference standard of expert, slice-by-slice adjustment of a SI threshold to quantitate extent of LGE. In contrast, FWHM-based segmentation is highly reproducible but provides a systematic under-representation of total LGE burden versus expert opinion. Consensus opinion regarding the preferred LGE segmentation methodology is required to facilitate the clinical translation of LGE quantification in the risk stratification of patients with HCM.

## Abbreviations

CMR: Cardiovascular magnetic resonance
HCM: Hypertrophic cardiomyopathy
LV: Left ventricular
LGE: Late gadolinium enhancement
STRM: Signal threshold versus reference mean
FWHM: Full width at half maximum
OAT: Otsu auto threshold
SI: Signal intensity
ICC: Intraclass correlation coefficient
ROI: Region of interest
SCD: Sudden cardiac death
